# Breast Cancer Treatment Practices in Elderly Women in a Community Hospital

**DOI:** 10.4061/2011/467906

**Published:** 2011-11-17

**Authors:** Hua Wang, Awinder P. Singh, Serena A. St. Luce, Alan R. Go

**Affiliations:** Department of Surgery, The Brooklyn Hospital Center, 121 Dekalb Avenue, Brooklyn, NY 11201, USA

## Abstract

*Background*. Elderly women with breast cancer are considered underdiagnosed and undertreated, and this adversely affects their overall survival. *Methods*. A total of 393 female breast cancer patients aged 70 years and older, diagnosed within the years 1989–1999, were identified from the tumor registry of The Brooklyn Hospital Center. Comparisons between the 3 different subgroups 70–74, 75–79, and 80 years and older were made using the Pearson Chi Square test. *Results*. Lumpectomy was performed in 42% of all patients, while mastectomy was done in 46% of patients. Adjuvant therapy such as chemotherapy, radiation therapy, and hormonal therapy were done in 12%, 25%, and 38%, respectively. Forty-seven percent of patients with positive lymph nodes received chemotherapy. Eighty-six percent of patients who were estrogen receptor-positive received adjuvant hormonal therapy. Overall five-year survival was only 14% for the ≥80 age group, compared to that of 32% and 35% for the 70–74 and the 75–79 age groups, respectively. *Conclusions*. Surgery was performed in majority of these patients, about half received lumpectomy, the other half mastectomy. Adjuvant therapies were frequently excluded, with only hormonal therapy being the most commonly used. Overall five-year survival is significantly worse in patients ≥80 years with breast cancer.

## 1. Introduction

Breast cancer is still the most common cause of death for women in the United States. Interestingly, we see that as the population ages, we likewise see an increase in the number of patients diagnosed with breast cancer. Increasing age remains the greatest risk factor in developing the disease. It is estimated that by 2030, 20% of Americans will be over the age of 65. Studies have shown that around 50% of patients with breast cancer are those older than 65 years of age and that approximately 35% are older than 70 years [1, 2]. Most women who die of breast cancer are over the age of 65 [3, 4]. Yet despite this information, it is observed in a number of studies that elderly patients do not receive the standard treatment when compared to their younger counterparts [5, 6]. And at the heart of this dilemma is that as patients advance in age, the incidence of cancer increases, but so does significant medical comorbidities. And it is the latter that may significantly affect treatment options in most patients. However, a number of studies have cited that age alone was a determining factor in the type of treatment a patient with breast cancer would receive [7, 8].

Studies collectively show that older patients with breast cancer are more likely to be undertreated compared to younger patients [9, 10]. To date, there is no consensus as to what the best treatment is for older patients [9]. Part of it is because older breast cancer patients are not very well represented in clinical trials [11]. There are also suggestions that treatment should include consideration of nonbreast-cancer-specific survival, functional status, and patient and family preference (goals of care). In terms of life expectancy, data from the National Vital Statistics Reports from the Centers for Disease Control and Prevention (CDC) released in 2003 have shown that females at age 70 have an average life expectancy of 15 years if they are relatively healthy and 7 years for those over 85 years old [12]. The care for an elderly breast cancer patient is indeed very complicated. Thus, treatment of an elderly patient should be multidisciplinary, possibly requiring the expertise of a primary care physician, a geriatrician, a psychiatrist, a surgeon, and a medical and radiation oncologist. 

Screening is likewise very controversial in the elderly population. There are very few retrospective and prospective studies looking specifically at cancer screening in the elderly. The US Preventive Services Task Force (USPSTF) recommends screening mammography, with or without clinical breast examination (CBE), every 1-2 years for women aged 40 and older [13]. Despite this, however, mammography was noted to be used 72% as adjunct to clinical examination in these older women and only around 30%–38% in women over 80 with occult disease [14]. These suggest that mammography was not largely used for screening purposes in the elderly patient with breast cancer [14].

Knowing that around 77% of deaths attributed to breast cancer are in women older than 55 years of age, it is truly interesting to note that there is an observed age-related difference in treatment and screening for breast cancer. The study intend to determine whether treatment of and screening for breast cancer in the elderly in a community hospital is appropriate. Improved treatment of older patients with breast cancer is of paramount importance if we are to properly address the growing number of cases and deaths due to breast cancer. 

## 2. Methods

The investigators identified the patients in the study using the American College of Surgeons Committee on Cancer-accredited tumor registry database of The Brooklyn Hospital Center. Patients between the years 1989–1999 were included in the study if they were older than 70 years old at the time of breast cancer diagnosis. The patients were then subsequently divided into three age subgroups: 70–74, 75–79, and ≥80, roughly corresponding to the age categories used in the geriatric population—the young old (often 65–74 years), the middle old (75–85 years), and the very oldest old (≥95 years). The following information for each subgroup were gathered from the registry: size, grade, histologic type of tumor, complete staging, estrogen receptor status, type of surgical procedure done, type of adjuvant therapy given, and survival data 5 years after diagnosis. The Pearson Chi-square test was used to compare patient characteristics and types of treatment between patient groups. A *P* value of 0.05 was designated as being significant. The protocol was approved by The Brooklyn Hospital Center's Institutional Review Board (IRB).

## 3. Results

There were a total of 393 female patients (age over 70 years) treated for breast cancer at the Brooklyn Hospital center between January 1989 and December 1999 that were divided into 3 different subgroups. There were 151 patients (38%) in the 70–74 years old group, 101 patients (27%) in the 75–79 years old groups, and 141 patients (36%) in the >80 years old group. Majority of our patient population was non-Hispanic blacks in all age groups (63%, 65%, and 52%), followed by Hispanics (26%), then white non-Hispanic (11%) and Asians (3%). The characteristics of the patients identified through the tumor registry are outlined in Table 1. Information on whether the patients presented with a palpable breast mass or were diagnosed based on mammography, as well as information on the patients' functional status and comorbidities were not available in the tumor registry database. There was no significant difference in the tumor size for all age groups (median size was 3.0 cm). Only 7% of all patients have a tumor size of <1.0 cm (nonpalpable). Thirty-nine percent of patients diagnosed were designated as having Stage II disease. Stage IV disease accounted for 3% of total cases. Ductal carcinoma was still the most common histologic type demonstrated in all age groups (69%, 67%, and 62%). Tumor grade was not identified in roughly half (43%) of these patients. Of those with tumor grading, moderately differentiated tumors were found to be most predominant. Complete staging was done in 71% of patients. It was noted to be omitted more in women older than 80, but was not statistically significant (*P* ≤ 0.20). About forty-three percent of tumors expressed estrogen receptors. However, about 46% of tumors had unknown estrogen receptor status. Similarly, only 36% of tumors had known positive progesterone status and was unknown in 46%. Surgery was not performed in about 11% of the patients (*P* ≤ 1). Breast conservation surgery (lumpectomy) was performed in about 42% of all patients, while mastectomy was done in 46% of patients. Of the patients who did not undergo surgery, it was not indicated in the tumor registry database as to whether they were not candidates or whether the patients had refused the procedure. Adjuvant therapy such as chemotherapy, radiation therapy, and hormonal therapy were done in 12%, 25%, and 38%, respectively. The use of chemotherapy was noted to have no significant difference in the three age groups. The same observation was noted for radiation and hormonal therapy with *P* values of ≤0.10 and ≤1, respectively. However, survival data after five years of diagnosis showed significant difference between the 75–79 and ≥80 age groups (*P* ≤ 0.001) as well as that of the 70–74 and the ≥80 age groups (*P* ≤ 0.001). Overall five-year survival was only 14% for the ≥80 age group, compared to that of 32% and 35% for the 70–74 and the 75–79 age groups, respectively. No significant difference was noted between the 70–74 and the 75–79 age groups in terms of five-year survival (*P* ≤ 1).

The type of therapy, if any, in addition to either mastectomy or lumpectomy was likewise analyzed from the tumor registry database and presented in Table 2. For patients who underwent mastectomy, there was note of a trend to offer adjuvant treatment to the younger cohort (70–74 versus ≥80 years old). However, the difference was not statistically significant (*P* ≤ 0.20). Chemotherapy and radiation therapy were given more to younger patients. That of hormonal therapy was noted to be the same. For those patients who underwent lumpectomy, the opposite trend was observed. Patients who were older were given more adjuvant treatment than the younger age group although again, the difference did not have statistical significance. Of note is that there were no patients given chemotherapy in those who had lumpectomy in the 70–74 age group, whereas about 20% received such therapy in the 80 and older group (*P* ≤ 0.01). 

The results in Table 3 showed that 47% of patients found to have positive lymph nodes received some form of chemotherapy either with a single agent or multiple agents. About 55% of patients in the 70–74 years age group received chemotherapy as opposed to only 35% in those 80 years old and greater. However, this difference was not significant (*P* < 1). There were 113 patients with positive margins of resection. Of those, 87% received radiation therapy. However, there was a significant difference in the number of patients who received radiation therapy in the 70–74 compared to that in the ≥80 age group (*P* ≤ 0.025). In patients with hormone receptor positive status, 84% received hormone therapy in the 70–74 age group and about 90% in the ≥80 age group.

## 4. Discussion

Previous studies have suggested that age was the strongest predictor of lesser treatment [3, 4] and that was what we sought to validate in the study. Our data have shown that there are observable differences in the different parameters measured among the subgroups and that there was a trend for the older cohort to be undertreated compared to the younger cohorts, but most of these observations were not deemed statistically significant. 

A notable difference was that of survival after five years of diagnosis. Significant differences were noted between the 80 and older age group and the 70–74 and 75–79 age groups. However, whether or not age alone in this case is the strongest predictor for survival cannot be fully determined. But besides age, perhaps the findings also imply that survival is impacted by other parameters besides the tumor and patient characteristics identified in the study and that perhaps functional status and comorbidities play a role. Previous studies in other cancer researches have shown that central to the decision making in the elderly is the concept of life expectancy [15, 16]. It is true that breast cancer increases with age. At the same time, as the age increases, the risk of death from causes besides breast cancer also increases [17]. Then, there is variability within an age cohort, with frail 70 year olds and robust 80 year olds. It is, therefore, significant to consider the patient's functional status and comorbidities in determining maximum treatment benefits for patients. It has been shown, for example, in cancer patients that poor functional status correlates with worse outcomes regardless of the type of cancer [18]. In weighing the benefits and risks of treatment therefore, breast cancer-specific prognosis, nonbreast-cancer-specific prognosis, and treatment-related toxicities should be accounted for. The National Comprehensive Cancer Network [19] has recommended the inclusion of the use of a geriatric assessment tool in developing care plans for the elderly cancer patient. Whether the assumption therefore, that perhaps functional status and comorbidities have contributed largely to the difference in survival cannot be determined as information was not available in the tumor registry. 

Surgery is still the mainstay of treatment for patients with early stage cancer. It has been shown that breast conservation surgery which involves lumpectomy with or without subsequent axillary dissection, followed by irradiation, has equal efficacy to more extensive surgical options such as mastectomy. However, current data suggests that elderly women are less likely to be offered breast conservation surgery than their younger counterparts (25% versus 42%) [20, 21]. Our study has shown that in this institution, mastectomy and lumpectomy were performed at almost the same frequency although adjuvant therapy was omitted in most cases. 

Staging was done for majority of patients (71%). There was no difference between age groups (*P* < 0.20) although younger patients were staged more than the 80 years and older cohort. Again, whether this was because of age alone cannot be determined. 

Still regardless of age, infiltrating ductal carcinoma is the most common histologic subtype as demonstrated in our study as well. Older patients have also been noted to have a greater frequency of tumors with more indolent histologies and overall more favorable biologic tumor profile. Older women are likely to express estrogen and progesterone receptors, which improves prognosis. This also makes them candidates for adjuvant hormonal therapy [22, 23]. In our study, majority of the patients with positive hormone receptor status received hormonal therapy. As suggested by prior studies, treatments that offer less toxicity are offered to patients with advanced age. Hormonal therapy which carries less risks and toxicity compared to chemotherapy is more likely to be offered and given to elderly patients with breast cancer. 

With regard to chemotherapy, cytotoxic drugs most commonly used in this setting like cyclophosphamide, methotrexate, and 5-fluorouracil represent the second line of systemic therapeutic agents available for women with breast cancer. There is paucity of data when it comes to women over the age of 70. In the Early Breast Cancer Trials Collaborative Group, it was shown that chemotherapy did not offer any advantage in survival for women over the age of 70 [24]. However, part of the limitation of this study was that there were very few cohorts over age 75 and that dosing was much less compared to the younger patient cohorts. Our study have shown that chemotherapy was not generally offered to our study cohort and although there was no statistical difference, there was note of a trend to give less chemotherapy as the patient gets older (see Table 3). This may stem from issues of toxicity of these agents and also by suggestions of less advantage as presented in previous trials. 

Radiation therapy is well tolerated in the elderly. It decreases ipsilateral breast cancer recurrence after breast conservation surgery and chest wall recurrence after a mastectomy. Our study showed that in patients 80 years and older, radiation therapy was given less than that in the 70–74 year old cohort (see Table 1), which is reasonable given numerous data that merely show a decrease in local recurrence but ultimately had no effect on survival [25, 26]. A previous study by Fyles et al. [27] showed that despite reduced risk of local (7.7 to 0.6%  *P* < 0.001) and axillary (2.5 to 0.5%  *P* < 0.49) recurrence, there was no significant difference in survival. The study of Ragaz et al. [28] demonstrated the same trend of decrease in locoregional recurrence but no advantage in decreasing distant metastasis or improving survival. It may be reasonable, therefore, to offer radiation therapy to patients who may benefit most such as those with life expectancy greater than 5 years, those with large tumors, positive lymph nodes, or negative hormone receptors.

## 5. Conclusions

Treatment of breast cancer in the elderly patient in a community setting, which in this case is The Brooklyn Hospital Center, has shown that there is a trend to having undertreatment with advancing age. It was likewise demonstrated that despite similar patient and tumor characteristics, the five-year survival significantly differed among the different age cohorts. To validate whether this is due to age alone, data on comorbidities and the patients' functional status should also be determined. It would also be significant to determine whether the patients recorded as not having received therapy were actually offered but refused, or never offered because of various reasons at all, as that would demonstrate more clearly the treatment practices as it relates to age and other patient characteristics.

## Figures and Tables

**Table 1:** Patient characteristics (1989–1999).

Characteristic	Age			Total	*P* Value
	70–74	75–79	≥80		
Patients, number (%)	151 (38)	101 (27)	141 (36)	393 (100)	NA

Race, number (%)					*P* ≤ 0.001 S
Black, non-Hispanic	95 (63)	66 (65)	73 (52)	234 (59)	
Asian	0 (0)	5 (5)	7 (5)	12 (3)	
White, non-Hispanic	39 (26)	2 (2)	2 (1)	43 (11)	
Hispanic	17 (12)	28 (28)	59 (42)	104 (26)	

Size of tumor, median ± standard deviation, cm	3.0 ± 2.7	2.5 ± 2.0	3.0 ± 1.96	NA	NA

Size of tumor, number (%)					NA
<1 cm (nonpalpable)	8 (5)	7 (7)	12 (9)	27 (7)	
≥1 cm (palpable)	126 (84)	82 (80)	113 (80)	321 (82)	
Unknown	16 (11)	13 (13)	16 (11)	45 (11)	

Stage, number (%)					*P* ≤ 0.20 NS
0	10	13	7	30 (8)	
1	38	22	33	93 (24)	
2	59	39	54	152 (39)	
3	23	9	24	56 (14)	
4	20	16	24	60 (15)	
Unknown	1	2	8	11 (3)	

Histologic type, number (%)					*P* ≤ 1 NS
Ductal	104 (69)	68 (67)	88 (62)	260 (66)	
Lobular	4 (3)	4 (4)	10 (7)	18 (4)	
Papillary	2 (1)	1 (1)	1 (1)	4 (1)	
Other	41 (27)	28 (28)	42 (30)	111 (28)	

Grade, number (%)					*P* ≤ 1 NS
1 (well differentiated)	7 (5)	6 (6)	6 (4)	19 (5)	
2 (moderately differentiated)	48 (32)	26 (26)	52 (37)	126 (32)	
3 (poorly differentiated)	24 (16)	13 (13)	20 (14)	57 (14)	
Unknown	72 (48)	55 (54)	63 (45)	190 (43)	

Estrogen receptor, number (%)					*P* ≤ 0.20 NS
Positive	69 (46)	44 (44)	58 (41)	171 (43)	
Negative	14 (9)	21 (21)	13 (9)	48 (12)	
Unknown	68 (45)	48 (47)	70 (50)	186 (47)	

Progesterone receptor, number (%)					*P* ≤ 1 NS
Positive	63 (42)	32 (32)	45 (32)	140 (36)	
Negative	24 (16)	21 (21)	26 (18)	71 (18)	
Unknown	64 (42)	48 (47)	70 (50)	182 (46)	

Surgical Procedure, number (%)					*P* ≤ 1 NS
Mastectomy	66 (44)	50 (49)	65 (46)	181 (46)	
Lumpectomy	67(44)	42 (41)	60 (42)	168 (42)	
No surgery	18 (12)	9 (9)	16 (11)	43 (11)	

Chemotherapy, number (%)					*P* ≤ 0.20 NS
Yes	22 (14)	12 (12)	11 (8)	45 (12)	
No	129 (85)	89 (88)	130 (92)	348 (88)	

Radiation therapy, number (%)					*P* ≤ 0.10 NS
Yes	47 (31)	21 (21)	30 (21)	98 (25)	
No	104 (69)	80 (79)	111 (79)	295 (75)	

Hormonal therapy, number (%)					*P* ≤ 1 NS
Yes	58 (39)	38 (38)	52 (37)	148 (38)	
No	93 (61)	63 (62)	89 (63)	245 (62)	

Had complete staging, number (%)					*P* ≤ 0.20 NS
Yes	115 (76)	73 (72)	93 (66)	281 (71)	
No	36 (24)	28 (28)	48 (34)	112 (28)	

Alive at 5 years after initial contact, number (%)					*P* ≤ 0.001 S
Yes	49 (32)	35 (35)	20 (14)	104 (26)	
No	102 (67)	66 (65)	121 (86)	289 (73)	

Abbreviation: NA: not applicable; NS: not significant; S: significant.

**Table 2:** Type of surgery and adjuvant treatment (1989–1999).

Type	Age			Total	*P* value
	70–74	75–79	≥80		
Had mastectomy, number (%)	66 (44)	50 (49)	65 (46)	181 (46)	*P* ≤ 0.20 NS
Alone	28 (42)	29 (58)	37 (57)		
With any adjuvant treatment	38 (58)	21(42)	28 (33)		
With radiation therapy	9 (14)	2 (4)	2 (3)		
With chemotherapy	7 (11)	4 (8)	3 (5)		
With hormonal therapy	22 (33)	15 (30)	23 (35)		

Had lumpectomy, number (%)	67 (44)	42 (41)	60 (42)	168 (42)	*P* ≤ 0.01 S
Alone	24 (36)	7 (17)	14 (23)		
With any adjuvant treatment	43 (64)	35 (83)	46 (77)		
With radiation therapy	22 (33)	12 (29)	15 (25)		
With chemotherapy	0 (0)	3 (7)	12 (20)		
With hormonal therapy	21 (31)	20 (48)	19 (32)		

Abbreviation: NA: not applicable; NS: not significant; S: significant.

**Table 3:** Indications for adjuvant therapy and rates of treatment (1989–1999).

Indication	Age			Total	*P* value
	70–74	75–79	≥80		
Positive lymph nodes, number	40	25	31	96	NA

Received chemotherapy, number (%)					*P* ≤ 1 NS
Yes	22 (55)	12 (48)	11 (35)	45 (47)	
No	18 (45)	13 (52)	20 (64)	51 (53)	

Positive margins of resection, number	47	27	44	113	NA

Received radiation therapy, number (%)					*P* ≤ 0.05 S
Yes	42 (89)	21 (78)	30 (68)	98 (87)	
No	5 (11)	6 (22)	14 (32)	25 (13)	

Progesterone receptor positive, number (%)	63 (42)	32 (32)	45 (32)	140	NA

Estrogen receptor positive, number (%)	69 (46)	44 (43)	58 (41)	171	NA

Received hormonal therapy, number (%)					*P* ≤ 1 NS
Yes	58 (84)	38 (86)	52 (90)	148 (86)	
No	11 (16)	6 (14)	6 (10)	23 (14)	

Abbreviation: NA: not applicable; NS: not significant; S: significant.
